# Galectin-1 Regulates Tissue Exit of Specific Dendritic Cell Populations*

**DOI:** 10.1074/jbc.M115.644799

**Published:** 2015-07-27

**Authors:** Sandra Thiemann, Jeanette H. Man, Margaret H. Chang, Benhur Lee, Linda G. Baum

**Affiliations:** From the Departments of ‡ Pathology and Laboratory Medicine and; § Microbiology, Immunology, and Molecular Genetics, David Geffen School of Medicine at UCLA, Los Angeles, California 90095 and; the ¶ Department of Microbiology, Icahn School of Medicine at Mount Sinai, New York, New York 10029

**Keywords:** dendritic cell, endothelial cell, extracellular matrix, galectin, inflammation, migration

## Abstract

During inflammation, dendritic cells emigrate from inflamed tissue across the lymphatic endothelium into the lymphatic vasculature and travel to regional lymph nodes to initiate immune responses. However, the processes that regulate dendritic cell tissue egress and migration across the lymphatic endothelium are not well defined. The mammalian lectin galectin-1 is highly expressed by vascular endothelial cells in inflamed tissue and has been shown to regulate immune cell tissue entry into inflamed tissue. Here, we show that galectin-1 is also highly expressed by human lymphatic endothelial cells, and deposition of galectin-1 in extracellular matrix selectively regulates migration of specific human dendritic cell subsets. The presence of galectin-1 inhibits migration of immunogenic dendritic cells through the extracellular matrix and across lymphatic endothelial cells, but it has no effect on migration of tolerogenic dendritic cells. The major galectin-1 counter-receptor on both dendritic cell populations is the cell surface mucin CD43; differential core 2 *O-*glycosylation of CD43 between immunogenic dendritic cells and tolerogenic dendritic cells appears to contribute to the differential effect of galectin-1 on migration. Binding of galectin-1 to immunogenic dendritic cells reduces phosphorylation and activity of the protein-tyrosine kinase Pyk2, an effect that may also contribute to reduced migration of this subset. In a murine lymphedema model, galectin-1^−/−^ animals had increased numbers of migratory dendritic cells in draining lymph nodes, specifically dendritic cells with an immunogenic phenotype. These findings define a novel role for galectin-1 in inhibiting tissue emigration of immunogenic, but not tolerogenic, dendritic cells, providing an additional mechanism by which galectin-1 can dampen immune responses.

## Teaser

**Background:** Galectins regulate immune cell migration.

**Results:** Galectin-1 inhibits immunogenic, but not tolerogenic, dendritic cell migration through clustering glycans on CD43.

**Conclusion:** Galectin-1 clustering of CD43 on immunogenic dendritic cells is a novel mechanism controlling tissue exit of different dendritic cell populations.

**Significance:** Inhibition of immunogenic dendritic cell tissue exit is a novel function for galectin-1 in dampening immune responses.

## Introduction

Effective adaptive immune responses require immune cells to infiltrate damaged tissue, acquire and process antigen, and migrate from inflamed tissue through the lymphatic vasculature to draining lymph nodes. To promote immune cell infiltration from the bloodstream into damaged tissue, vascular endothelial cells (VECs)^4^ and immune cells in the blood up-regulate expression of adhesion molecules and their cognate ligands (1, 2). Monocytes are a critical immune cell population recruited to inflamed tissue; once in tissue, monocytes differentiate into immature dendritic cells (DCs) and then, depending on the combination of stimuli in the tissue, mature into distinct DC subsets that have distinct phenotypes and functions (3–7). Mature DCs can exit inflamed tissue by migrating through the extracellular matrix (ECM) and across lymphatic endothelial cells (LECs) in a basolateral-to-apical direction, entering the lymphatic vasculature and migrating through lymphatic channels to draining lymph nodes, where the DCs participate in promoting or controlling adaptive immune responses (8–10). Like VECs, LECs play a critical role in shaping immune responses by controlling transport of immune cells and soluble antigen from inflamed tissue to lymph nodes (11–18). Although some of the molecular interactions between LECs and immune cells have been described (17–19), the majority of molecular interactions regulating the exit of different DC subsets from tissue and into the lymphatic vasculature remains unknown.

Once DCs reach draining lymph nodes, the nature of the adaptive immune response that ensues is significantly shaped by the types of DCs that left the tissue and arrived at the lymph nodes. Mature, immunogenic DCs (iDCs) process antigens and can initiate and prime pro-inflammatory T cell responses (20). Conversely, semi-mature, tolerogenic DCs (tDCs) also process antigen (21–24), but they suppress pro-inflammatory T cell responses by promoting generation of regulatory T cells, inhibiting T cell proliferation, and inducing T cell death (20, 23–25). Various stimuli have been shown to render DCs either immunogenic or tolerogenic *in vitro*. For example, iDCs develop in the presence of inflammatory cytokines, such as tumor necrosis factor-α (TNF-α), toll-like receptor ligands such as lipopolysaccharide (LPS), or CD40 ligation (26–32). In contrast, contact with inflamed lymphatic endothelium, apoptotic cell debris, vitamin D, corticosteroids, histamine, or the carbohydrate-binding protein galectin (gal)-1 can all render DCs tolerogenic (25, 33–37). Although it is known that different inflammatory stimuli can shift the balance between iDCs and tDCs and thus determine the nature and amplitude of T cell activation, it is not well understood how the balance of iDCs and tDCs in the draining lymph node is controlled.

In inflamed tissue, expression of the carbohydrate-binding protein galectin-1 is increased in vascular endothelial cells (38, 39). Galectin-1 has been shown to inhibit entry of leukocytes from the bloodstream into tissues at sites of inflammation *in vivo* and to retard the migration of T cells through extracellular matrix *in vitro* (40–44). However, the role of galectins in influencing the exit of leukocytes from tissues and into draining lymphatic vasculature is not well understood. Two reports have suggested a role for galectins in regulating migration of dermal DCs to draining lymph nodes under inflammatory conditions. Using a dermal inflammation model, Hsu *et al.* (45) reported reduced numbers of migrating dermal DCs in the draining lymph nodes of galectin-3^−/−^ mice compared with wild type, implying that galectin-3 promotes migration of dermal DCs from inflamed tissue to draining nodes. Using the same dermal inflammation model, we demonstrated that injection of recombinant galectin-1 prior to the inflammatory stimulus resulted in increased DC numbers in draining lymph nodes in MRL-*fas* mice, promoting maturation of tolerogenic rather than immunogenic DCs (35). Although both galectin-3 and galectin-1 may regulate DC exit from inflamed tissue, it is not clear how migration of immunogenic *versus* tolerogenic DC subsets is affected by the presence of galectins in tissue. Moreover, as galectins in VECs are important for regulation of leukocyte entry into tissues, galectins produced by LECs may similarly influence leukocyte exit from tissues. Although a previous report described expression of galectin-8 by LECs (46), we found that LECs also express abundant galectin-1. Moreover, galectin-1 expression by LECs remained robust after treatment with inflammatory cytokines. Thus, we sought to determine whether galectin-1 could regulate iDC and tDC migration through the matrix and tissue exit across LECs and to identify DC cell surface glycoproteins that interact with galectin-1 to regulate tissue exit of distinct DC subsets.

## Experimental Procedures

### 

#### 

##### Mice

Galectin-1 null (galectin-1^−/−^) animals (47) backcrossed onto the C57BL/6 background for 13+ generations (48) were provided by Drs. R. J. Singh and M. C. Miceli (David Geffen School of Medicine, UCLA). Wild type C57BL/6J mice were purchased from The Jackson Laboratory (Bar Harbor, ME). Animals were housed under guidelines set by the National Institutes of Health, and experiments were conducted in accordance with the Chancellor's Animal Research Committee (UCLA) and the Public Health Service Policy on Humane Care and Use of Laboratory Animals.

##### Human Tissue Samples

Anonymized, paraffin-embedded punch biopsies of human lymphedema skin were provided by the Translational Pathology Core Laboratory at UCLA (David Geffen School of Medicine, UCLA).

##### Cell Culture

Human dermal lymphatic endothelial cells (HMCV-DLyAd-Der Lym Endo) were purchased from Lonza (Walkersville) and maintained in EGM^TM^-2MV medium (Lonza) as described (49). To observe changes in galectin expression under inflammatory conditions, LECs were treated for 48 h with 3 ng/ml TNF-α, 10 ng/ml Il-1α, or 10 ng/ml IFN-γ.

Human immature dendritic cells were differentiated from purified monocytes as described (36). Immature dendritic cells were matured by addition of 100 ng/ml lipopolysaccharide (LPS) or 20 μm recombinant human galectin-1 for the last 48 h of culture. Cells were washed twice in 1× PBS prior to use in migration assays.

##### Reagents and Antibodies

Recombinant human galectin-1 was produced as described previously (50). Reagents were obtained from the indicated suppliers as follows: BD BioCoat^TM^ Matrigel^TM^ Invasion Chambers, 8-μm pore size (BD Biosciences); recombinant human IL-4, GM-CSF, TNF-α, Il-1α, IFN-γ, and MIP-3β/CCL19 (PeproTech); CellTrace^TM^ carboxyfluorescein succinimidyl ester (CFSE) proliferation kit (Invitrogen); CD16/CD32 (mouse BD FC block^TM^, BD Biosciences); benzyl-2-acetoamido-2-deoxy-α-d-galactopyranoside (Bn-α-GalNAc) (Calbiochem); LightCycler® 480 SYBR Green I Master reagent (Roche Applied Science); hematoxylin (Vector Laboratories); 3,3′-dithiobis[sulfosuccinimidylpropionate] (DTSSP) (Thermo Scientific); phosphatase and protease inhibitor mixtures (Sigma); methylene blue (Sigma); 4′,6-diamidino-2-phenylindole (DAPI) (Invitrogen); protein G beads (Pierce); and enhanced chemiluminescence (ECL) detection kit (GE Healthcare).

The following antibodies were used: rabbit anti-human galectin-1 polyclonal antibody serum (pAb) (Strategic); rat anti-mouse galectin-3 antibody (clone M3/38) (BioLegend); mouse anti-human galectin-9 (Novus Biologicals); mouse anti-human podoplanin (clone D2-40) (Covance); mouse anti-human CD86-phycoerythrin (PE) (clone BU63) (Invitrogen); mouse anti-human CD40-PE (clone HB14) (BioLegend); mouse anti-human CD43 (clone 1D4) (MBL); mouse anti-human CD43 (clone DF-T1) (DakoCytomation). Isotype controls for anti-human monoclonal antibodies (mAb) are as follows: mouse IgG1, mouse IgG2a, mouse IgG2b (all mouse isotype controls were purchased from DakoCytomation); rat IgG2a (BioLegend); polyclonal rabbit serum (Gibco).

To analyze murine lymph node cells by flow cytometry, the following antibodies and corresponding isotype controls were used: rat anti-mouse B220-allophycocyanin (clone RA3-6B2); Armenian hamster anti-mouse CD11c-fluorescein isothiocyanate (FITC) (clone N418); rat anti-mouse CD86 (B7–2)-PE (clone GL1); rat anti-mouse CD83-PE (clone Michel-19); rat anti-mouse MHC class II-eFluor® 450 (clone M5/114.15.2); rat anti-mouse CD45RB-PE (clone C363.1A6); rat anti-mouse CD274-Brilliant Violet 605^TM^ (PD-L1, clone 10F9G2) (BioLegend) (all anti-mouse antibodies and isotype control antibodies were purchased at eBioscience unless otherwise noted); rabbit anti-phospho-Pyk2 (Y402) and rabbit anti-Pyk2 (Cell Signaling Technology); goat anti-rabbit IgG (H+L)-horseradish peroxidase (HRP) (Jackson ImmunoResearch).

##### Surgical Lymphedema Model

A surgical model was used to introduce lymphedema in the mouse tail in adult (10–12 weeks old) wild type C57BL/6 and galectin-1^−/−^ mice, as described previously (51, 52). Briefly, lymph stasis was induced in anesthetized animals by circumferential cauterization of the superficial dermal lymphatics 10 mm proximal to the tail base. Cauterization of the major lymphatic vessels was visualized by subcutaneous injection of a 10% methylene blue solution distal to the surgical incision. In control animals (sham animals), skin incision was performed with methylene blue injection but without lymphatic cautery ablation. Animals were euthanized at day 6 post-surgery. Cell suspensions from tail-draining inguinal lymph nodes (53) were analyzed by flow cytometry as described below.

##### Flow Cytometry Analysis

Human dendritic cells were incubated for 30 min on ice with the indicated fluorochrome-labeled antibodies or with the appropriate isotype controls and fixed in 4% paraformaldehyde (PFA) before flow cytometry analysis. Binding of galectin-1 was assessed by incubating human dendritic cells with 0.2 mg/ml biotinylated recombinant galectin-1 for 30 min on ice. The cells were then fixed in DTSSP at 0.2 mg/ml for 10 min at room temperature, and quenched by the addition of 100 μl of 1 m Tris, pH 7.5, for 15 min at room temperature. Galectin-1 binding was visualized with FITC-conjugated streptavidin (Jackson ImmunoResearch).

Single-cell suspensions from mouse inguinal lymph nodes were prepared. Nonantigen-specific binding of antibodies was blocked by incubating cells with antibodies against CD16/CD32 (Mouse BD FC block^TM^) prior to addition of staining antibodies. Cells were incubated for 30 min on ice with the indicated fluorochrome-labeled antibodies or with the appropriate isotype controls. Cells were washed in 3% FBS in PBS and fixed in 4% PFA for 20 min at 4 °C before flow cytometry analysis. All samples were analyzed on FACSCalibur or LSRFortessa flow cytometers (BD Biosciences) using FlowJo analysis software (Tree Star).

##### Cytokine Production Analysis

Tissue culture supernatant was collected from LPS- or galectin-1-matured dendritic cell cultures after 24 h of maturation and stored at −80 °C until analysis. Cytokine secretion was determined using human IL-10 (eBioscience) and IL-12 (BD Bioscience) sandwich ELISA kits.

##### Real Time Quantitative RT-PCR

cDNA was prepared from human DCs using the RNeasy mini kit (Qiagen) and the Super-Script III One-Step RT-PCR kit (Invitrogen). cDNA was amplified with specific primer sets using the LightCycler® 480 SYBR Green I Master reagent (Roche Applied Science) with the LightCycler® 480 (Roche Applied Science) and its detection software. The following primer sets were used: C2GnT-I, 5′-TTATTGTTTGAAATGCTGAGGACG-3′ (sense) and 5′-TAATGGTCAGTGTTTTAATGTCT-3′ (antisense) (54); 36B4, 5′-CCCGCTGCTGAACATGCT-3′ (sense) and 5′-TCGAACACCTGCTGGATGAC-3′ (antisense) (55). Relative expression of the gene of interest was normalized to relative expression of the housekeeping gene *36B4*.

##### In Vitro Dendritic Cell Migration Assay

*In vitro* dendritic cell migration assays were performed as described (49). Briefly, LECs were grown to confluence on the underside of Matrigel^TM^-covered transwell inserts (8 μm pore-size, BD Biosciences) before Matrigel^TM^ was saturated with 1× PBS or 20 μm recombinant galectin-1 in PBS. CFSE-labeled DC populations were seeded into the top of the transwell insert and incubated for 24 h before the number of migrated DCs in the bottom well was determined by counting the cells in the bottom and top wells in combination with flow cytometry (49). The percentage of migrating cells was calculated as the number of DCs in the bottom well/total number of DCs in the top and bottom wells. Data are shown as % migration in the presence of galectin-1 in Matrigel^TM^/% migration in the absence of galectin-1 in Matrigel^TM^.

##### Immunohistochemistry

3 × 10^5^ LECs were grown to >80% confluency on Lab-Tek® II chamber slides (Nalgene Nunc International) and fixed in 4% PFA for 10 min at room temperature. Slides were blocked with 1% bovine serum albumin (BSA) in PBS for 20 min at room temperature. pAbs against galectin-1 or polyclonal rabbit serum were diluted 1:1000 in 20% goat serum in 1% BSA in PBS, and cells were incubated in antibody dilutions overnight at 4 °C. After washing in PBS, fixed cells were incubated with FITC-conjugated goat anti-rabbit IgG diluted in PBS for 2 h at room temperature and washed in PBS. Cells were then stained with 300 nm DAPI stain (Invitrogen) for 5 min at room temperature, rinsed in PBS, and analyzed using an Olympus BX51 fluorescence microscope and Olympus DP2-BSW software (Olympus America Inc.).

To analyze human tissue, 6-μm serial sections of paraffin-embedded punch biopsies of human lymphedema skin were de-paraffinized and stained with pAb against galectin-1 or polyclonal rabbit serum. For podoplanin staining, mouse anti-human podoplanin (clone D2-40) was used at a 1:100 dilution as described above and detected with the anti-mouse DakoCytomation EnVision+^TM^ kit (Dako). Both stainings were visualized using a 3-amino-9-ethylcarbazole chromogenic substrate system (Enzo Life Sciences). All sections were counterstained with hematoxylin (Vector Laboratories) and analyzed using an Olympus BX51 fluorescence microscope and Olympus DP2-BSW software (Olympus America Inc.).

##### Immunoprecipitation and Western Blot

For identification of galectin-1 binding partners on DCs, cells were washed once in 0.5 m lactose/PBS and 1× PBS, surface-biotinylated as described (35, 56, 57), washed in 1× PBS, and treated with 20 μm galectin-1 for 1 h at 4 °C before fixation with DTSSP (58). Cells were lysed in 1% Nonidet P-40 lysis buffer with protease and phosphatase inhibitor mixtures (Sigma) as described (59). For immunoprecipitation, lysates were adjusted to 40 μg of total protein in 100 μl of lysis buffer and immunoprecipitated with 10 μl of rabbit anti-human galectin-1 antibody and 20 μl of protein G beads overnight at 4 °C. After immunoprecipitation, protein G beads plus precipitated protein were washed three times in lysis buffer with protease and phosphatase inhibitor mixtures and denatured in NuPAGE sample buffer and reducing agent (Invitrogen), and proteins were then separated on a 4–12% BisTris polyacrylamide gel in MOPS buffer before transfer to nitrocellulose membranes (GE Healthcare) for immunoblotting.

For analysis of Pyk2 phosphorylation after galectin-1 binding, DCs were harvested, washed in 0.5 m lactose/PBS and 1× PBS, resuspended in warm 1× PBS, and treated with 20 μm recombinant galectin-1 for the indicated times at 37 °C. After galectin-1 addition, cells were immediately placed on ice, pelleted, and lysed in RIPA buffer with 1% Triton X-100 plus protease and phosphatase inhibitors. 40 μg of total protein lysate were denatured in NuPAGE sample buffer and reducing agent (Invitrogen), and proteins were separated by SDS-PAGE and transferred to nitrocellulose membranes (GE Healthcare) for immunoblotting. Immunoblotting was performed with the indicated primary antibodies and corresponding HRP-coupled secondary antibodies. Antibody binding was visualized using ECL.

##### Inhibition of O-Glycan Elongation

1 × 10^6^ immature DCs were treated with 2 mm Bn-α-GalNAc in dimethyl sulfoxide (DMSO) or DMSO alone (control) for 4 days. DCs were matured with either LPS or galectin-1 (as described above) during the last 2 days of Bn-α-GalNAc treatment.

##### Confocal Microscopy

Untreated or Bn-α-GalNAc-treated DCs were collected, washed in 0.5 m lactose/PBS, and incubated with recombinant galectin-1 for 1 h at 37 °C. PBS-washed DCs were then fixed in 4% PFA at room temperature. After fixing, all staining steps were performed at room temperature. Cells were washed and incubated with blocking solution (5% BSA in PBS, 4 °C) for 20 min. All samples were incubated in primary antibody in blocking solution for 1 h, washed in PBS, incubated with secondary antibody in PBS for 1 h while protected from light, washed, incubated with the nuclear stain DAPI for 5 min, washed in PBS, and mounted on slides using Prolong Gold Antifade reagent (Invitrogen). Confocal images were taken on a Leica TCS-SP2 AOBS inverted confocal microscope (Mannheim, Germany) using Leica confocal software.

##### Statistical Analysis

Statistical significance was determined using the unpaired two-tailed Student's *t* test for single comparisons. *p* values smaller than 0.05 were considered statistically significant. All statistical analysis was performed using GraphPad Prism software (version 4).

## Results

### 

#### 

##### Human Dermal Lymphatic Endothelial Cells Express and Secrete Galectin-1

**FIGURE 1.**
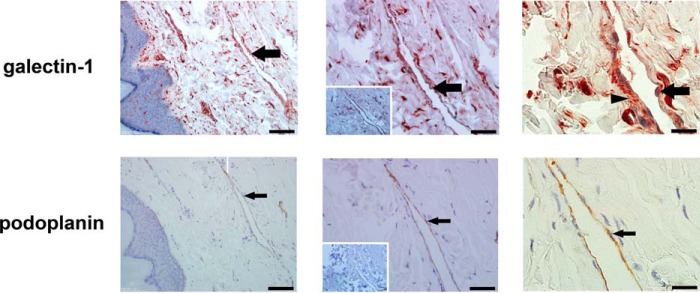
**Human lymphatic endothelial cells express and secrete galectin-1.** Sections of skin from lymphedema patients were stained with polyclonal antibody against galectin-1 (*top*) or monoclonal antibody to the human LEC marker podoplanin (*bottom*). Bound antibody was detected with the corresponding secondary antibody and visualized using a 3-amino-9-ethylcarbazole chromogenic substrate system. Sections were counterstained with hematoxylin. *Insets* (*middle column*) show control antibody staining. Dilated lymphatic vessels are lined by LECs expressing galectin-1 (*arrow, top*) and podoplanin (*arrow, bottom*). Data are representative of six independent tissue samples. Note that the distribution of galectin-1 on LECs appears more dispersed than that of podoplanin, suggesting the localization of secreted galectin-1 in extracellular matrix (*arrowhead*, *top right panel*). Magnification is as follows: ×20 (*left*), ×40 (*middle*), and ×100 (*right*). *Scale bar,* 100 μm (*left*), 50 μm (*middle*), and 20 μm (*right*).

Galectin-1 is expressed by human VECs, and VEC expression of galectin-1 is increased by inflammatory stimuli (38, 39). However, galectin-1 expression by LECs in inflamed human tissue *in vivo* has not been described. To characterize galectin-1 expression by LECs in inflamed tissue, we examined sections of lymphedematous human skin (Fig. 1). Sections were stained with pAb against human galectin-1 (Fig. 1, *top row*) or a mAb recognizing the LEC marker podoplanin (Fig. 1, *bottom row*) (60). In the dermis, galectin-1 was detected in endothelial cells (*arrow, top row*) lining dilated vessels; these same cells also expressed podoplanin (*arrow, bottom row*), confirming that the cells were LECs. In addition, there was abundant galectin-1 in the extracellular matrix surrounding lymphatic channels (*arrowhead, top right panel*). We also detected galectin-1 in infiltrating leukocytes in the dermis.

**FIGURE 2.**
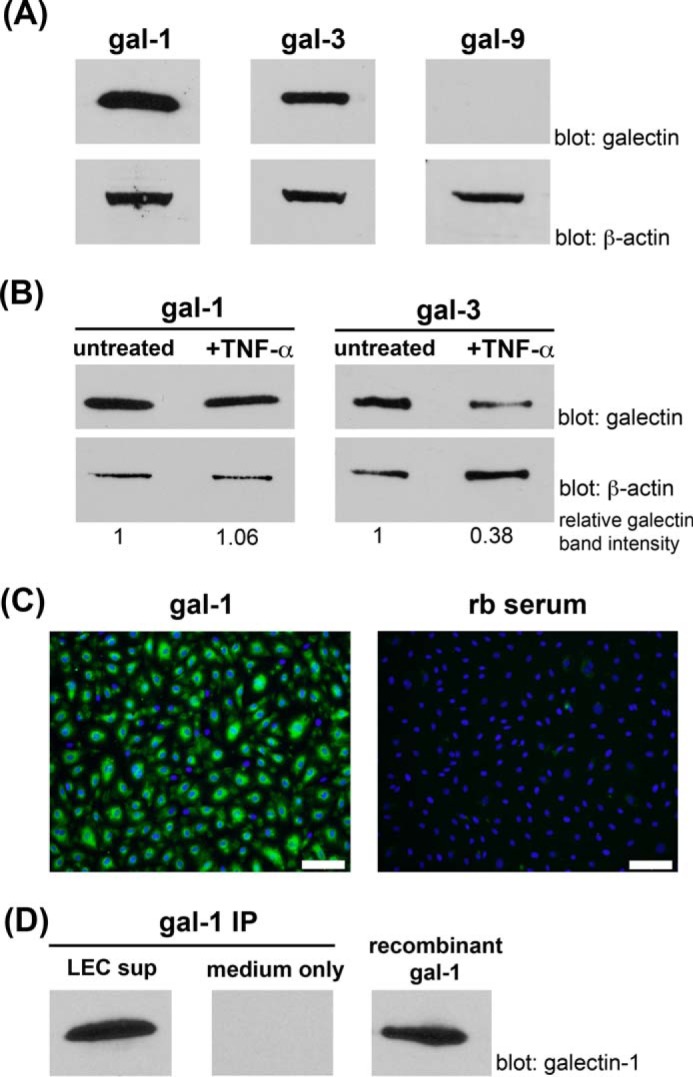
**Cultured human LECs express and secrete galectin-1.** *A,* primary human LECs were analyzed for expression of gal-1, gal-3, and gal-9 by immunoblot. *B,* primary human LECs were grown in the presence or absence of the inflammatory cytokine TNF-α for 24 h, and cell lysates were analyzed for expression of galectin-1 and galectin-3 by immunoblot. Densitometry quantification of the relative change in galectin expression is indicated at the bottom of the figure. Relative galectin band intensity was calculated for each sample as a ratio of the pixel intensity in the scanned galectin band over the β-actin band. The relative galectin band intensity of the untreated sample was assigned a value of 1, and the +TNF-α sample was displayed as a proportion of the untreated sample. Data are representative of four independent experiments. Galectin-1 expression remained abundant in TNF-α-treated LECs, although galectin-3 expression decreased significantly after TNF-α treatment. *C,* confluent LECs were fixed with 4% PFA, and surface galectin-1 was detected by immunofluorescence microscopy using pAb against galectin-1 and FITC-conjugated secondary antibody. Nuclei were visualized with DAPI staining. Galectin-1 is detectable on the cell surface indicating that galectin-1 secreted by LECs binds back to glycans on the LEC surface. *Scale bar,* 100 μm. *rb serum*, rabbit serum. *D,* serum-free medium supernatant from cultured human LECs (*LEC sup*) was collected, and galectin-1 protein was immunoprecipitated (*IP*) for detection by immunoblot. Abundant galectin-1 was detected in the LEC supernatant but not in LEC-free medium (*medium only*). Immunoprecipitation of galectin-1 from a control sample containing 125 ng of recombinant galectin-1 is shown for comparison (*right*).

We examined galectin expression in cultured primary human dermal LECs by immunoblot (Fig. 2*A*). Galectin-1 was abundantly expressed in LECs (about 0.76 pg/cell, ∼0.85% of total LEC protein, data not shown), similar to concentrations described in other tissues (61, 62). In contrast to VECs that express galectin-1, -3, -8, and -9 (39, 63), LECs express only galectin-1, -3 (Fig. 2*A*), and -8 (46) but not galectin-9 (Fig. 2*A*).

To examine galectin-1 expression in LECs under inflammatory conditions, we incubated LECs with the pro-inflammatory cytokine TNF-α (64) and analyzed cell lysates by immunoblot (Fig. 2*B*). Notably, galectin-1 was abundant in control LECs, and treatment with TNF-α did not appreciably change the level of galectin-1 expression. In contrast, when the same lysates were analyzed for another member of the galectin family, we observed that galectin-3 abundance decreased significantly in cells treated with TNF-α compared with controls. Similar results were obtained using additional pro-inflammatory stimuli, such as IL-1α and IFN-γ (data not shown).

Galectin-1 is a soluble protein that is secreted from the cell and can bind to cell surface glycoconjugates or to surrounding extracellular matrix glycoproteins (65–70). To determine whether dermal LECs secreted galectin-1, we first examined cell surface galectin-1. LECs were fixed with 4% PFA, and cell surface galectin-1 was detected by immunofluorescence microscopy (Fig. 2*C*). In addition, we examined serum-free medium supernatant from cultured human LECs (*LEC sup*) and detected secreted galectin-1 in the medium (Fig. 2*D*). Thus, LECs express and secrete galectin-1, which is both bound to the cell surface and released into the extracellular milieu.

##### Galectin-1 Regulates Human Dendritic Cell Migration through Extracellular Matrix and across LECs

**FIGURE 3.**
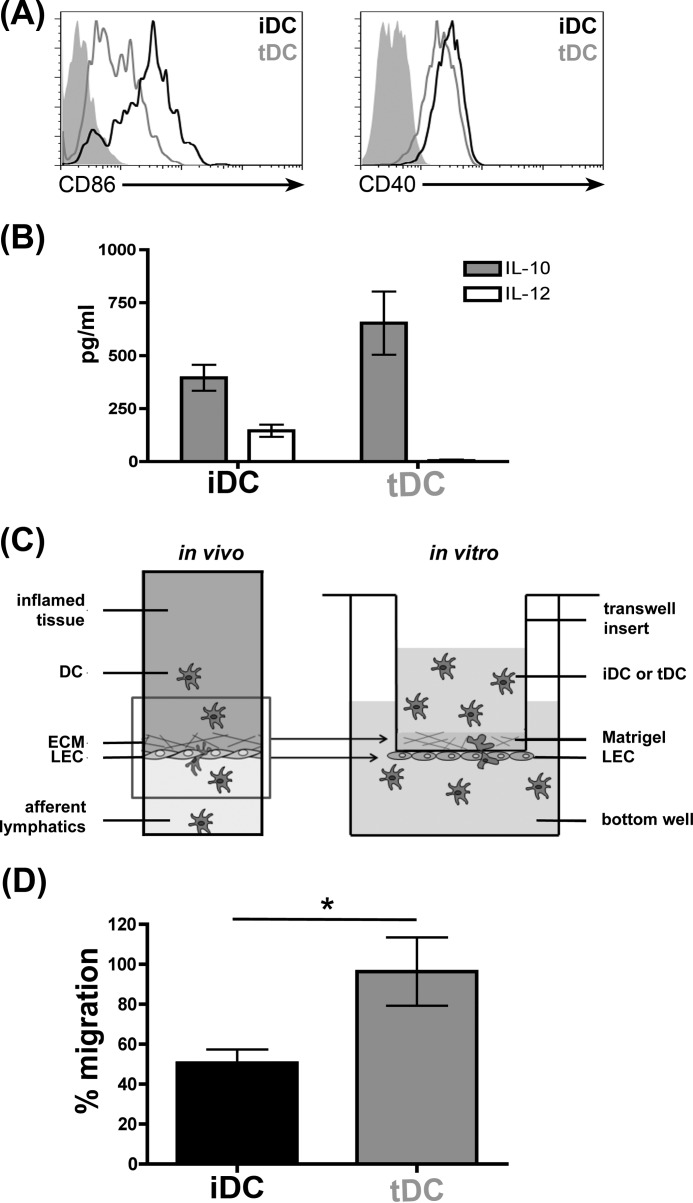
**iDCs and tDCs are distinct DC populations that differ in the ability to migrate through ECM and across LECs in the presence of galectin-1.** *A,* phenotypic analysis of iDCs and tDCs. iDCs and tDCs were analyzed for expression of the cell surface markers CD86 and CD40 by flow cytometry. Although iDCs expressed high levels of CD86 and CD40, expression was lower on tDCs. *Filled histograms* are isotype controls. *B,* secretion of IL-10 and IL-12 by iDCs and tDCs. Although iDCs secreted significant levels of both IL-10 and IL-12, tDCs only secreted IL-10. Data are combined from four independent donors each analyzed in duplicate ± S.E. *C, in vivo* model of DCs emigrating from inflamed tissue across LECs in the basolateral-to-apical direction (*left*). *In vitro* model of DC migration assay through matrix and across LECs (*right*). LECs were grown to confluency on the underside of Matrigel^TM^-covered transwell inserts, and Matrigel^TM^ was impregnated with recombinant galectin-1 or buffer alone. 5 × 10^4^ iDCs or tDCs labeled with CFSE were placed into the top chamber, and cells migrated to the bottom well (containing the chemoattractant MIP-3β) for 24 h. *D,* presence of galectin-1 inhibited migration of iDCs, but not tDCs, through the matrix and across LECs. Results are shown as % migration in the presence of galectin-1 in Matrigel^TM^/% migration in the absence of galectin-1 in Matrigel^TM^. Data are combined from four independent experiments ± S.E. *, *p* = 0.04.

We have previously found that galectin-1 secreted by VECs regulated T cell migration through ECM (44). Thus, we asked whether galectin-1 in ECM would affect DC migration through ECM and across LECs in a basolateral-to-apical direction; this path would mimic the egress of DCs from tissue into the lymphatic vasculature. As described above, distinct populations of iDCs and tDCs shape adaptive immune responses, and the differential migration of iDCs *versus* tDCs from inflamed tissue could affect subsequent immune responses in draining lymph nodes. To examine the migration of human iDCs and tDCs, iDCs and tDCs were generated *in vitro* from peripheral blood mononuclear cells as described previously (35, 36) and analyzed by flow cytometry to confirm maturation. As expected, iDCs expressed high levels of cell surface CD86 and CD40, although the relative expression of these cell surface markers was lower in tDCs (Fig. 3*A*) (33, 34, 36). Furthermore, iDCs secreted IL-10 and IL-12, although tDCs only secreted large quantities of IL-10, which has been shown to promote resolution of inflammation (Fig. 3*B*) (71).

To analyze the effect of galectin-1 on DC migration, we designed an *in vitro* migration assay that mimics *in vivo* DC tissue exit (Fig. 3*C*) (49). Briefly, LECs were grown as a confluent monolayer on a porous membrane on the underside of Matrigel^TM^-covered transwell inserts, so that the basal side of the LECs was proximal to the extracellular matrix (LEC transwell insert). CFSE-labeled iDCs or tDCs were placed in the upper chamber of the LEC transwell inserts, in the absence or presence of recombinant galectin-1 in the Matrigel^TM^. Although LECs secreted galectin-1 into tissue culture media (Fig. 2*D*) and into ECM when plated on Matrigel^TM^ directly (data not shown), we could only detect minimal galectin-1 secreted into the Matrigel^TM^ from LECs on the underside of the transwell membrane (data not shown), indicating that galectin-1 did not effectively cross the transwell membrane to bind to ECM glycans in Matrigel^TM^ inside the transwell insert. Thus, we added recombinant galectin-1 directly to Matrigel^TM^ to examine the effect of galectin-1 on DC migration (49). After 24 h, the number of CFSE-labeled DCs that migrated through the ECM and across LECs in a basolateral-to-apical direction and into the bottom well was calculated as a fraction of the total number of DCs added to each well.

In the absence of galectin-1, similar fractions of iDCs and tDCs migrated through Matrigel^TM^ and across the LEC monolayer into the bottom well in 24 h (data not shown). Moreover, addition of galectin-1 to Matrigel^TM^ had no appreciable effect on tDC migration as the percent of migrated cells in wells containing galectin-1 was approximately equal to the percent of migrated cells in the absence of galectin-1 (Fig. 3*D*). In contrast, we observed a significant decrease in iDC migration in the presence of galectin-1; iDC migration in the presence of galectin-1 was ∼50% of iDC migration in the absence of galectin-1 (Fig. 3*D*). Thus, galectin-1 in ECM selectively retarded the migration of iDCs but had no effect on tDC migration.

##### CD43 Is the Major Glycoprotein Counter-receptor for Galectin-1 on Human iDCs and tDCs

Galectins preferentially recognize *N*-acetyl-lactosamine (LacNAc) sequences that can be presented on *N*- or *O-*glycans (70, 72–74). Thus, galectins can bind to an array of cell surface glycoproteins that bear the preferred glycan ligands. However, it is clear that different galectins preferentially bind to only a subset of total cell surface LacNAc-bearing glycoproteins on various types of cells so that there are preferred glycoprotein counter-receptors for specific galectins on a particular cell type (72, 74–76). As galectin-1 retarded migration of iDCs but had no effect on tDC migration in the transwell assay (Fig. 3*D*), we asked whether the differential effect on migration that we observed was related to galectin-1 binding different glycoprotein receptors on the two DC subsets.

**FIGURE 4.**
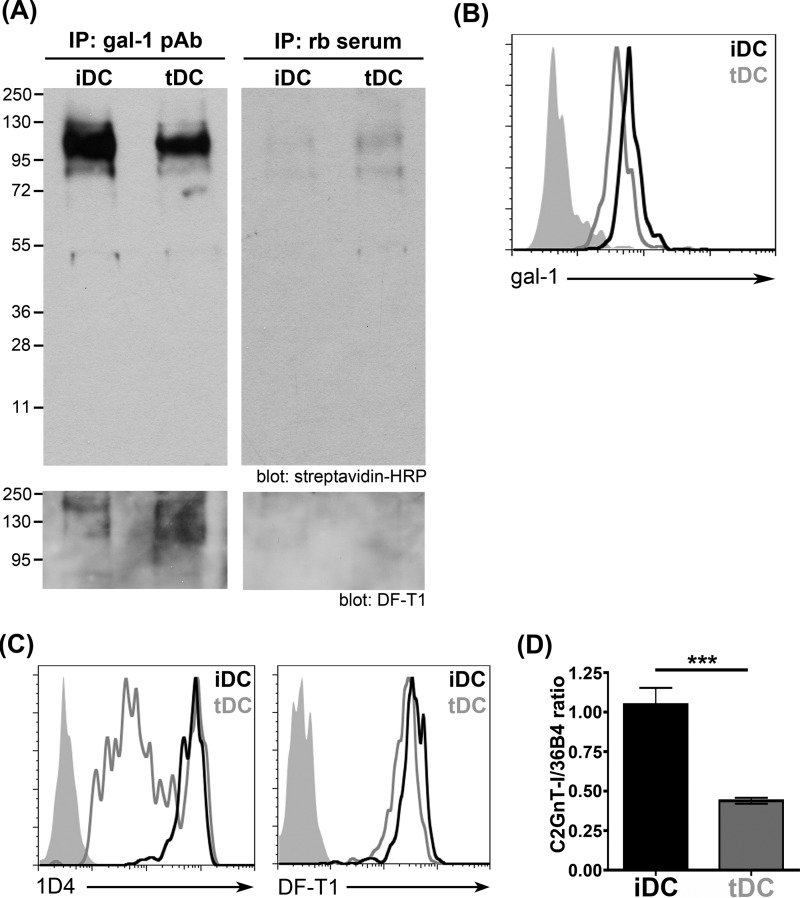
**Galectin-1 binds CD43 on iDCs and tDCs.** *A,* iDCs and tDCs were incubated with recombinant galectin-1 for 1 h at 4 °C. iDCs and tDCs were biotinylated, incubated with recombinant galectin-1 for 1 h at 4 °C, and fixed with DTSSP. After chemical cross-linking, galectin-1 plus bound cell surface counter-receptors were immunoprecipitated (*IP*) with rabbit anti-galectin-1 pAb. Control samples were immunoprecipitated with rabbit serum (*rb serum*). Immunoprecipitates were separated on a 4–12% BisTris polyacrylamide gel in MOPS buffer. Blots were probed with streptavidin-horseradish peroxidase (*HRP*), stripped, and re-probed with antibody against CD43. A single band of ∼115 kDa was detected in samples from both iDCs and tDCs that reacted with the pan-specific CD43 mAb DF-T1. *B,* iDCs bind slightly more exogenous galectin-1 than tDCs. Recombinant biotinylated galectin-1 was added to iDCs and tDCs at 4 °C, and bound galectin-1 was detected with FITC-conjugated streptavidin. Data shown are representative of four independent experiments. *Filled histogram* is the rabbit serum (*rb serum*) control staining. *C,* differences in CD43 core 2 *O-*glycosylation on iDCs and tDCs were identified by flow cytometry using mAb 1D4 (*left*) that recognizes core 2 *O-*glycans on human CD43. Although iDCs and tDCs expressed equivalent levels of total CD43, detected by mAb DF-T1 (*right*), iDCs express significantly more CD43 decorated with core 2 *O-*glycans compared with tDCs. *Filled histograms* are isotype controls. *D,* β(1,6)-*N*-acetylglucosaminyltransferase (*C2GnT-I*) mRNA expression was analyzed by quantitative RT-PCR and normalized to expression of the housekeeping gene *36B4*. Results are from three independent experiments ± S.E. ***, *p* = 0.001. iDCs express >3-fold more β(1,6)-*N*-acetylglucosaminyltransferase mRNA than tDCs, consistent with the higher expression of core 2 *O-*glycan modified CD43 on iDCs compared with tDCs in *C*.

To identify glycoprotein counter-receptors for galectin-1 on iDCs and tDCs, we used an immunoprecipitation approach that we have previously used to isolate galectin-1 binding partners on immature DCs and on T cells (35, 56, 57). Total surface proteins on iDCs and tDCs were biotinylated before addition of recombinant human galectin-1. After washing, cells were lysed, and galectin-1 plus associated glycoproteins were immunoprecipitated with anti-galectin-1 pAb bound to protein G beads. Precipitates were separated by SDS-PAGE, and biotinylated cell surface glycoproteins that precipitated with galectin-1 were identified by streptavidin binding. As shown in Fig. 4*A* (*top left panel*), the precipitated complexes generated from both iDCs and tDCs contained one predominant species of ∼130 kDa. Although the same band was detected in immunoprecipitates from both iDCs and tDCs, the protein band in the iDC lane was slightly more broad compared with the band in the tDC lane, potentially indicating increased glycan complexity or abundance. Immunoblotting with mAb against human CD43 (clone DF-T1) revealed the primary binding partner for galectin-1 in both DC subsets to be CD43 (Fig. 4*A*, *bottom panel*).

As CD43 appeared to be the predominant counter-receptor for galectin-1 on both iDCs and tDCs, yet galectin-1 retarded iDC but not tDC migration (Fig. 3*D*), we reasoned that the differential effect of galectin-1 on iDC *versus* tDC migration might result either from a difference in the abundance of glycoprotein receptors available on iDCs and tDCs to bind galectin-1 or, alternatively, a difference in the glycans present on CD43 on iDCs and tDCs. To examine total galectin-1 binding to the two DC populations, recombinant biotinylated galectin-1 was bound to the cell surface of iDCs and tDCs, and bound galectin-1 was detected with FITC-labeled streptavidin. As shown in Fig. 4*B*, both iDCs and tDCs bound abundant galectin-1, although iDCs bound slightly more exogenous galectin-1 compared with tDCs. Thus, we asked whether differential glycosylation of CD43 on iDCs and tDCs could contribute to the differential effect of galectin-1 on migration of these two DC subsets.

CD43 is a large cell surface mucin decorated with 70–80 *O-*glycans (77–79). The *O-*glycans on CD43 can be either core 1- or core 2-type *O-*glycans; as core 2 *O-*glycans present LacNAc sequences preferentially recognized by galectin-1, galectin-1 binds CD43 decorated with core 2 *O-*glycans with higher avidity compared with CD43 decorated with core 1 *O-*glycans (80, 81). To examine the relative level of expression of CD43 decorated with core 2 *O-*glycans on the two DC subsets, we used the mAb 1D4 that specifically recognizes CD43 bearing core 2 *O-*glycans (Fig. 4*C*, *left*) (54, 82). iDCs comprised one relatively homogeneous population of cells with relatively high 1D4 binding. In contrast, overall binding of 1D4 to tDCs was reduced compared with iDCs, and there was heterogeneous staining of the tDCs with the 1D4 mAb. Interestingly, iDCs and tDCs expressed roughly the same level of CD43 protein on the cell surface (measured by binding of mAb DF-T1 that recognizes all CD43 glycoforms (83)) (Fig. 4*C*, *right*). iDCs also had robust expression of mRNA encoding core 2 β(1,6)-*N*-acetylglucosaminyltransferase (C2GnT-I), the enzyme that creates core 2 *O-*glycans on leukocytes (84–86), whereas tDC expression of C2GnT-I was 3-fold lower compared with iDCs (Fig. 4*D*). As iDCs have higher, more uniform expression of CD43 decorated with core 2 *O-*glycans (Fig. 4*C*, *left*), these data suggested that the differential effect of galectin-1 on migration of iDCs *versus* tDCs was related to expression of core 2 *O-*glycans on CD43 on these two cell types.

**FIGURE 5.**
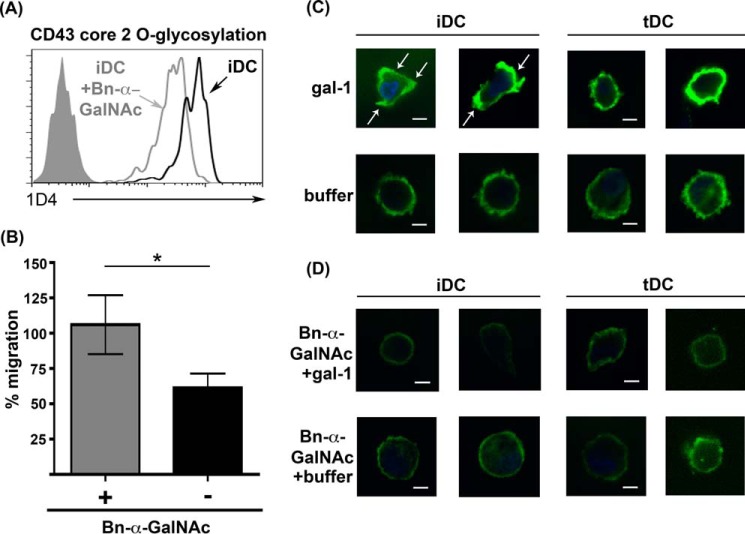
**Inhibition of *O-*glycosylation reverses galectin-1 inhibition of iDC migration and prevents CD43 clustering on iDCs in the presence of galectin-1.** *A,* iDCs were treated with 2 mm Bn-α-GalNAc (*iDC* + *Bn*-α-*GalNAc*) or with buffer control (*iDC*). Expression of CD43 bearing core 2 *O-*glycans was analyzed by flow cytometry with the mAb 1D4. *Filled histogram* is the isotype control. Treatment with Bn-α-GalNAc decreased binding of mAb 1D4. *B, in vitro* migration assays were performed as described in Fig. 3*C*. Bn-α-GalNAc-treated and untreated iDCs were migrated across Matrigel^TM^ plus LECs in the presence or absence of recombinant galectin-1 in Matrigel^TM^. Inhibition of *O-*glycan elongation reversed the inhibitory effect of galectin-1 on iDC migration. Results are shown as % migration in the presence of galectin-1 in Matrigel^TM^/% migration in the absence of galectin-1 in Matrigel^TM^. Results are representative of three independent experiments. Three replicate samples ± S.D. are shown for each data point, *, *p* = 0.019. *C*, iDCs or tDCs were treated with recombinant galectin-1 for 1 h at 37 °C. After fixation, samples were processed for confocal microscopy using antibody against CD43 (mAb DF-T1) and FITC-coupled secondary antibody. Galectin-1 clustered CD43 on iDCs (*white arrows*) but not on tDCs. Nuclei were visualized with DAPI. Two different images from different experiments are shown for each condition. *Scale bar,* 5 μm. *D,* iDCs or tDCs were treated with 2 mm Bn-α-GalNAc before recombinant galectin-1 was added for 1 h at 37 °C. Samples were processed as above. Inhibition of *O-*glycan elongation with Bn-α-GalNAc abrogated galectin-1-induced clustering of CD43 on iDCs. Two different images from different experiments are shown for each condition. *Scale bar,* 5 μm.

Because core 2 *O-*glycans have lactosamine sequences that are preferred ligands for galectin-1, we asked whether inhibition of *O-*glycan elongation with the pharmacological inhibitor Bn-α-GalNAc (87) would affect iDC migration in the presence of galectin-1. iDCs were treated with 2 mm Bn-α-GalNAc for 4 days during maturation, and a decrease in core 2 *O-*glycan expression on CD43 was confirmed by flow cytometry with the 1D4 mAb (Fig. 5*A*). We then asked whether Bn-α-GalNAc treatment affected iDC migration, using the *in vitro* migration assay (Fig. 3*C*) in the absence or presence of galectin-1 added to Matrigel^TM^. Bn-α-GalNAc treatment did not affect iDC migration in the absence of galectin-1 in Matrigel^TM^ (data not shown). Although the presence of galectin-1 in Matrigel^TM^ inhibited iDC migration, Bn-α-GalNAc treatment of iDCs reversed this inhibitory effect, increasing the fraction of iDCs that migrated through the Matrigel^TM^ insert and across LECs to the bottom well (Fig. 5*B*). These data indicated that *O-*glycans on iDCs are important for galectin-1-induced inhibition of iDC migration. tDC migration, however, was unaffected by Bn-α-GalNAc treatment regardless of the presence of galectin-1 in Matrigel^TM^ (data not shown).

Galectin-1 binding to cell surface glycoprotein receptors has been shown to regulate intracellular signaling events through clustering receptors via multivalent galectin-glycan lattices (35, 88, 89). Specifically, we have found that binding of galectin-1 clusters CD43 on the surface of DCs and T cells (35, 44, 57) and that this clustering inhibits T cell migration through the ECM (44). Thus, we asked whether galectin-1 differentially clustered CD43 on iDCs and tDCs, and whether CD43 clustering was dependent on *O-*glycans. iDCs and tDCs were incubated with recombinant galectin-1 or buffer control, fixed, stained with anti-CD43 (mAb DF-T1), and analyzed by confocal microscopy. Strikingly, in the presence of galectin-1, CD43 on iDCs clustered in distinct patches on the cell surface (Fig. 5*C*, *white arrows, top left*). In contrast, in either the presence or absence of galectin-1, CD43 on tDCs was evenly distributed on the cell surface (Fig. 5*C*, *right*), indicating that, although galectin-1 can bind to tDCs and precipitate CD43, galectin-1 binding does not result in CD43 clustering on tDCs.

To interrogate the role of *O-*glycans in galectin-1-mediated clustering of CD43 on iDCs, iDCs and tDCs were treated with Bn-α-GalNAc, as described above, prior to the addition of galectin-1. On tDCs, CD43 was evenly distributed on the cell surface in the absence or presence of Bn-α-GalNAc (Fig. 5*D*, *right*). However, when iDCs were treated with Bn-α-GalNAc, galectin-1 failed to cluster CD43 (Fig. 5*D*, *top left*); the even distribution of CD43 on Bn-α-GalNAc-treated iDCs resembled that seen for tDCs, indicating that *O-*glycans on CD43 are critical for the formation of galectin-1-CD43 lattices on iDCs.

##### Differential Pyk2 Phosphorylation in iDCs and tDCs Correlates with Differences in Migration

Although signaling pathways that regulate migration of neutrophils and lymphocytes in and out of tissue are well described, relatively little is known about the intracellular signaling pathways that regulate DC migration. Recent work has shown that, in murine iDCs, tyrosine phosphorylation of Pyk2 regulates DC migration *in vitro* and *in vivo* (90). Inhibition of Pyk2 enzymatic activity or reduction in Pyk2 expression resulted in reduced iDC migration. Thus, we asked whether differential phosphorylation of Pyk2 in iDCs *versus* tDCs was related to the differences in galectin-1-mediated regulation of migration of these two DC subsets.

**FIGURE 6.**
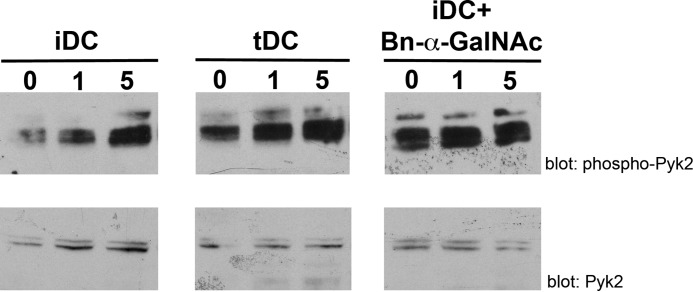
***O-*Glycan-dependent binding of galectin-1 regulates Pyk2 phosphorylation.** iDCs, tDCs, or iDCs treated with 2 mm Bn-α-GalNAc were treated with 20 μm recombinant galectin-1 for 0, 1, or 5 min. Cell lysates were analyzed for phosphorylated Pyk2 and total Pyk2 protein expression. Phosphorylation of Pyk2 was lower in iDCs treated with galectin-1 compared with tDCs at the same time points. Bn-α-GalNAc treatment of iDCs prior to addition of galectin-1 increased Pyk2 phosphorylation.

To examine Pyk2 phosphorylation, iDCs and tDCs were exposed to recombinant galectin-1 for 0, 1, and 5 min, and Pyk2 phosphorylation was analyzed by immunoblot. As shown in Fig. 6, binding of galectin-1 resulted in an increase in Pyk2 phosphorylation in both tDCs and iDCs, but Pyk2 in iDCs was relatively hypophosphorylated compared with tDCs at all time points. Bn-α-GalNAc treatment of iDCs to reduce *O-*glycan elongation resulted in increased Pyk2 phosphorylation of iDCs to levels comparable with tDCs, suggesting that galectin-1 binding to *O-*glycans on iDCs signal upstream to regulate Pyk2 phosphorylation and thus activity. Given the requirement of Pyk2 activity for iDC migration (90), these data implied that galectin-1 binding to iDCs impaired iDC migration by regulation of the Pyk2 pathway.

##### Increased iDC Migration to Draining Lymph Nodes in Galectin-1^−/−^ Mice

**FIGURE 7.**
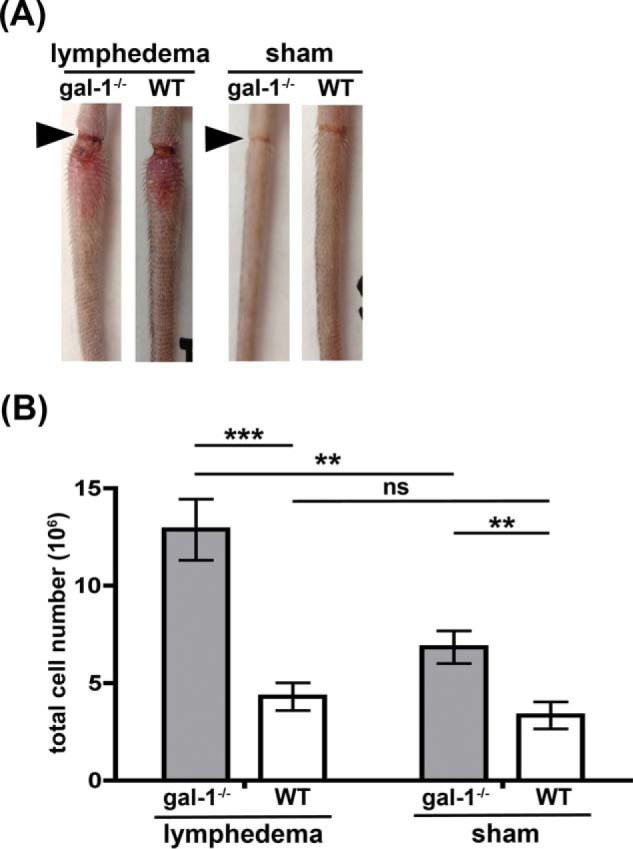
**Absence of galectin-1 *in vivo* increases the number of cells in regional lymph nodes.** Superficial dermal lymphatics in the tail were cauterized in galectin-1^−/−^ (*gal-1*^−/−^) and C57BL/6 (*WT*) animals to induce lymphedema. Animals were analyzed at day 6 post-surgery. *A,* edema adjacent to the incision site (*arrowhead*) was visible in animals when lymphatics were cauterized (lymphedema), while sham-treated animals (skin incision alone) had no edema. *B,* cell suspensions from tail-draining lymph nodes were counted to determine the total number of cells in the node. Lymph nodes from galectin-1^−/−^ were larger (data not shown) and had more cells compared with lymph nodes from WT mice, and the difference was more pronounced in animals with lymphedema. Data are combined results of *n* >10 animals per group and five independent experiments ± S.E. ***, *p* < 0.0001; **, *p* = 0.034; *ns*, *p* > 0.2.

The *in vitro* migration assays shown in Figs. 3*D* and 5*B* examined the directional migration of DCs through ECM and across LECs in a basolateral-to-apical direction, to mimic the path DCs would take when migrating from tissue into lymphatics to traffic to draining lymph nodes. To ask whether galectin-1 regulates iDC migration from tissue across lymphatic endothelium and into draining lymphatics *in vivo*, we examined migration of dermal iDCs from the tail to regional, tail-draining lymph nodes using a murine surgical lymphedema model. In this model, inflammation and lymph stasis are initiated by a superficial surgical incision in the tail with cauterization of superficial dermal lymphatic vessels, resulting in tissue damage, edema, and subsequent migration of inflammatory cells from damaged tissue through the remaining lymphatics to the tail-draining lymph nodes (51–53). As shown in Fig. 7*A*, edema developed in the portion of the tail adjacent to the incision in both wild type (WT) and galectin-1^−/−^ mice (*left*), but not in sham-treated animals that only had the superficial incision but no lymphatic cauterization (*right*). When draining lymph nodes were examined (data not shown), the lymph nodes from galectin-1^−/−^ mice were larger than those in control animals; a modest difference in lymph node size between wild type and galectin-1^−/−^ mice was noted in sham-treated animals, but there was a dramatic increase in the size of nodes from galectin-1^−/−^ mice compared with wild type mice in the animals with lymphedema. As shown in Fig. 7*B*, in both sham-treated and lymphedema mice, lymph nodes from galectin-1^−/−^ mice had more total cells than those from wild type mice. The increase in total cell numbers in lymph nodes from lymphedema animals was most pronounced in galectin-1^−/−^ mice; we observed a 3-fold increase in the total cell numbers in lymph nodes of galectin-1^−/−^ mice compared with wild type mice.

**FIGURE 8.**
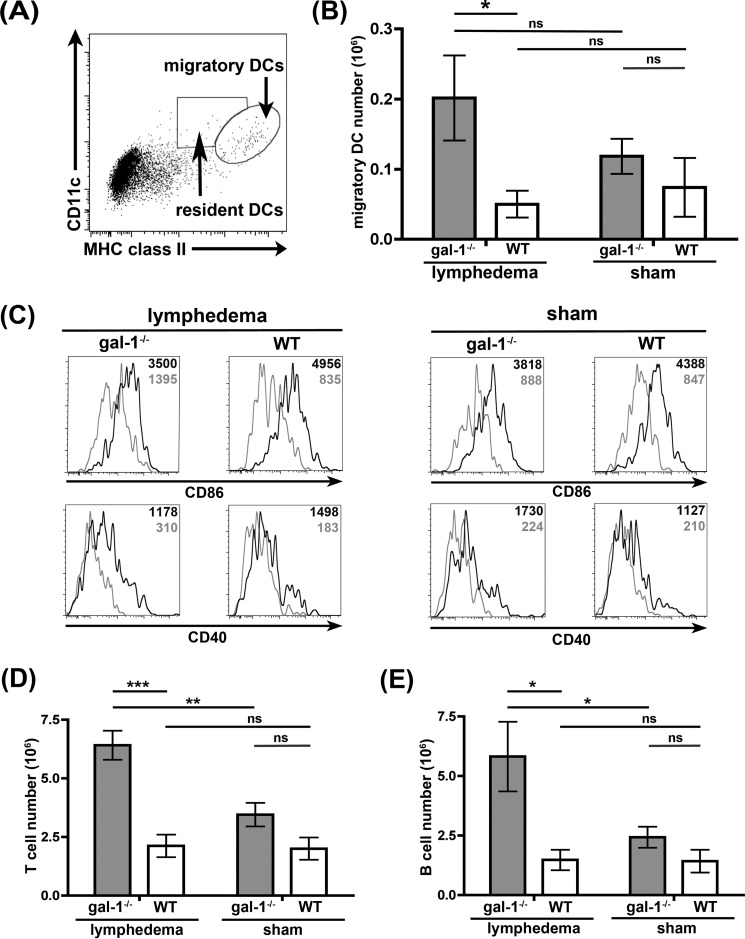
**Increased numbers of migratory iDCs in draining lymph nodes in galectin-1^−/−^ mice.** *A*, identification of migratory and resident DCs. B220^−^ lymph node cells were analyzed by flow cytometry for expression of CD11c and MHC class II as a marker for migratory DCs (CD11c^high^/MHC class II^high^). *B*, total number of migratory CD11c^high^/MHC class II^high^ cells is shown for each group. Note the significant increase in migratory DCs in galectin-1^−/−^ mice with lymphedema compared with control animals. Data are combined results of at least 10 animals per group and five independent experiments ± S.E. *, *p* = 0.0263; *ns, p* > 0.2. *C*, migratory DCs in draining lymph nodes have an immunogenic DC phenotype. When expression of CD86 and CD40 was compared on resident (*gray line*) and migratory (*black line*) DCs in the same animal, migratory DCs were CD86^high^, CD40^high^, as described for iDCs. Mean fluorescence intensities for CD86 or CD40 detection in both populations are indicated as *black* (migratory DCs) and *gray* (resident DCs) *numbers* inside the *histograms. D*, increased numbers of T cells in lymph nodes, detected by CD3 staining, from galectin-1^−/−^ mice with lymphedema. Data are combined results of at least 10 animals per group and five independent experiments ± S.E. ***, *p* = 0.001; **, *p* = 0.0023, *ns*, *p* > 0.05. *E*, increased numbers of B cells, detected by CD19 staining, from galectin-1^−/−^ mice with lymphedema. Data are combined results of at least 10 animals per group and five independent experiments ± S.E. *, *p* < 0.03; *ns, p* > 0.2. Note that the increase in migratory DCs (*B*) correlates with the increased T and B cell numbers in lymph nodes of galectin-1^−/−^ animals.

To determine the cellular composition of the lymph nodes, DCs and T and B cells in the tail-draining lymph nodes were analyzed by flow cytometry. Mature dendritic cells are end-stage cells (7); thus, an increase in DCs in draining lymph nodes most likely results from increased migration of DCs from tissues through lymphatics to the regional lymph nodes. The total migratory DC population in the nodes was identified by expression of high levels of CD11c and MHC class II (Fig. 8*A*), whereas resident DCs in the lymph nodes are CD11c^high^/MHC class II^intermediate^ (Fig. 8*A*) (3). Quantification of the two DC populations in the lymph nodes demonstrated an increase in dendritic cells that had migrated to the lymph nodes in lymphedema galectin-1^−/−^ mice, relative to wild type mice. Moreover, as expected from a model in which surgery and superficial lymphatic cauterization resulted in tissue damage and inflammation (51, 52, 91), the migratory DCs that were present had the phenotype of iDCs, *i.e.* CD86^high^, CD40^high^ (Fig. 8*C*, *black lines* in *histograms*) and CD45RB^high^, PD-L1^low^ (data not shown), when compared with resident DCs that were CD86^low^, CD40^low^ (Fig. 8*C*, *gray lines* in *histograms*). Thus, in the absence of galectin-1, we observed a dramatic increase in the number of iDCs migrating from damaged tissue to regional lymph nodes.

We also observed an increase in the absolute numbers of T and B cells in the lymph nodes in galectin-1^−/−^ mice compared with control mice (Fig. 8, *D* and *E*), most pronounced in the lymphedema animals. Inflammation and migration of antigen-loaded iDCs from draining tissues to regional lymph nodes will stimulate proliferation of T and B cells within the nodes, implying that the increase in T and B cells could result from increased local proliferation driven by the increased numbers of dermal iDCs arriving in the lymph nodes of the galectin-1 ^−/−^ mice.

## Discussion

Dynamic changes in the expression of galectin-1 and other galectin family members in specific tissues and cell types regulate a wide range of leukocyte activities. In particular, galectin-1 has been shown to regulate DC differentiation, signaling, and migration (33, 36, 37, 92–94). Previous work described that galectin-1 inhibits migration of lymphocytes and neutrophils into inflamed tissues (95–97). However, the processes that regulate leukocyte exit from inflamed tissues are still not well understood, and the role of galectin-1 in leukocyte emigration from tissue is unknown. This study describes a novel role for galectin-1 in regulating migration and tissue exit of different terminally matured DC populations. As DCs are the primary antigen-presenting cell type that migrates to the lymph node to drive T and B cell proliferation, these data describe a new mechanism by which galectin-1 can regulate immune responses.

Each cell type expresses a unique repertoire of galectins, and constitutive *versus* inducible regulation of galectin expression is also cell type-specific. Human VECs express galectin-1, -3, -8, and -9 (39), whereas human dermal LECs express galectin-1, -3, and -8 but not galectin-9 (Fig. 2, *A* and *B*) (46). Inflammatory stimuli increase galectin-1 expression by VECs (38, 39), whereas the constitutively high expression of galectin-1 by LECs appeared unchanged by inflammatory stimuli; in contrast, LEC expression of galectin-3 was reduced after treatment with inflammatory cytokines, such as TNF-α (Fig. 2*B* and data not shown). Although our analysis of human dermal lymphatic endothelial cells both *in vivo* and *in vitro* (Fig. 1 and 2) demonstrated robust galectin-1 expression, it is important to note that the concentration of other molecules that regulate migration, such as CCL21, is variable among lymphatics in different anatomic locations (98), and it is not yet known whether galectin-1 expression by lymphatic endothelial cells is also variable depending on the anatomic location of the LECs.

In addition to regulation of galectin expression in various anatomic compartments, the biological outcome of a galectin binding to a target cell is dependent on the glycome of the target cell, *i.e.* both the array of glycan structures that are expressed by the cell and the different glycoproteins on the cell surface that are decorated with those glycans. For example, galectin-1 kills specific subpopulations of human and murine thymocytes, depending on the glycan ligands expressed by the different subsets of cells (56, 99, 100), and galectin-1 kills Th1 but not Th2 CD4 T cells, again because of differential T cell glycosylation (99, 101, 102). In this study, we observed that galectin-1 in ECM differentially affected migration of two populations of mature human DCs, iDCs and tDCs. Galectin-1 in Matrigel^TM^ retarded migration of iDCs that were matured by exposure to the bacterial component LPS, although galectin-1 in Matrigel^TM^ had no effect on migration of tDCs matured by exposure to galectin-1 (Fig. 3*D*). Interestingly, we found that different tDC populations, matured by different “tolerogenic” signals, exhibited different migration behavior. Although tDCs matured by exposure to galectin-1 demonstrated robust migration, we observed no migration of tDCs matured by exposure to dexamethasone in this assay, regardless of the presence of galectin-1 (data not shown). Although published literature has described a common phenotype for tDCs matured by a wide variety of tolerogenic signals, including inflamed lymphatic endothelium (34), apoptotic cell debris, vitamin D, corticosteroids, histamine, and galectin-1 (24, 33, 36, 103–105), our results suggest that tDCs matured by different tolerogenic stimuli may have different functional activities.

As described above, differential effects of the same galectin on different cell types can be due to differential glycosylation, affecting either the abundance or the type of glycan ligands present on the target cell. We found that iDCs expressed abundant core 2 *O-*glycans on the transmembrane mucin CD43, although core 2 *O-*glycan expression was variable on tDCs (Fig. 4*C*). Expression of mRNA encoding the C2GnT-I glycosyltransferase was also higher in iDCs compared with tDCs (Fig. 4*D*). Pharmacological inhibition of *O-*glycan elongation reversed the effect of galectin-1 on retarding iDC migration (Fig. 5*B*), supporting a model in which expression of core 2 *O-*glycans on CD43 is a primary determinant of the effect of galectin-1 on DC migration. The presence of core 2 *O-*glycans also regulates susceptibility to other galectin-1 responses, such as galectin-1-induced T cell death (54, 81, 99, 101, 106, 107); galectin-1-mediated clustering of CD45 on the T cell plasma membrane is dependent on expression of core 2 *O-*glycans, and CD45 clustering and subsequent inhibition of the cytoplasmic phosphatase activity of CD45 are required for T cells to die after binding galectin-1 (108). Core 2 *O-*glycans on B lymphoma cells are important for binding of another galectin, galectin-3, that inhibits B lymphoma cell death in response to chemotherapeutic agents (59). Alternatively, in other lymphocyte populations, such as thymocytes and Th1 *versus* Th2 CD4 T cells, the ST6Gal1 sialyltransferase is a critical glycosyltransferase that regulates cell susceptibility to galectin-1; addition of terminal α2,6-linked sialic acids due to ST6Gal1 activity masks the lactosamine glycan ligands preferentially recognized by galectin-1 and confers resistance to galectin-1-induced cell death (101, 109). Although there are several reports of differential glycosylation regulating T cell responses to galectins (110) and differential effects of glycosylation of human DC subsets on galectin-1 binding (111, 112), to our knowledge this is the first report of differential glycosylation of DC populations regulating the cellular response to a galectin.

Appropriate glycosylation and presentation of glycan ligands for galectins on the cell surface are important not just for galectin binding to single glycoprotein receptors, but for multivalent galectins binding multivalent glycan ligands to create cell surface lattices or glycoprotein clusters. Lattice formation of glycoprotein receptors with different galectins has been shown to regulate T cell receptor and cytokine receptor signaling (113–116), regulate lipid raft stability (117, 118), target glycoproteins to specific delivery sites in polarized cells (119–121), and determine cell surface residency time of nutrient transporters (122, 123). These effects can result from increased retention of cell surface proteins on the plasma membrane (122, 124, 125) or from altered signal intensity due to receptor clustering (35, 36, 48, 56, 59, 113, 115, 117, 124, 350), and they can be homotypic clusters of a single glycoprotein receptor or heterotypic clusters of different glycoprotein receptors (35, 37, 44, 57, 99, 109).

Although prior studies have identified several glycoprotein receptors for galectin-1 on T cells and immature dendritic cells (35, 37, 56, 57, 127), we identified only one predominant glycoprotein receptor for galectin-1 on human iDCs and tDCs, the cell surface mucin CD43 (Fig. 4*A*). CD43 is also a receptor for galectin-1 on T cells (56, 57). Although not essential for galectin-1-induced T cell death (81), we found that galectin-1 inhibition of T cell migration involved galectin-1 clustering of CD43 at the leading edge of the migrating T cell (44), consistent with the observation that redistribution of CD43 from the leading edge to the trailing edge is required for T cell migration (128). Little is known about the roles of specific cell surface glycoproteins in regulating DC migration; however, it is striking that galectin-1 binding clustered CD43 on iDCs but did not cluster CD43 on tDCs (Fig. 5*C*). Importantly, reducing *O-*glycan elongation with Bn-α-GalNAc abrogated galectin-1 clustering of CD43 on iDCs (Fig. 5*D*, *top left panels*) and also reversed the galectin-1-mediated inhibition of iDC migration (Fig. 5*B*), indicating that clustering of CD43 via galectin-1 binding to core 2 *O-*glycans is important for retarding iDC migration. This suggests a novel role for CD43 in regulating DC migration.

Although “traffic signals,” such as chemokines, that regulate DC migration have been studied in detail, relatively little is known about the intracellular signaling pathways that control DC migration. Recent work has shown that reduced Pyk2 expression or pharmacological inhibition of Pyk2 resulted in reduced iDC migration (90). Reduced iDC migration in the presence of galectin-1 correlated with reduced overall Pyk2 phosphorylation in these cells (Fig. 6). These results indicate that galectin-1 binding to iDCs reduces Pyk2 activity and that this may contribute to the inhibition of iDC migration.

In addition to regulating iDC migration *in vitro*, galectin-1 also appears to regulate iDC migration *in vivo* in a surgical lymphedema model (Figs. 7 and 8). Several previous studies have described altered immune function in galectin-1^−/−^ mice, specifically increased CD4 Th1-mediated and CD8-mediated responses in various disease models (101, 129, 130). Galectin-1^−/−^ mice are more susceptible to collagen-induced arthritis and to experimental autoimmune encephalitis (101, 124, 131), and galectin-1 retards T cell trafficking to inflamed tissue (95, 96). Our data demonstrate that, even in the absence of lymphedema-induced tissue damage, lymph nodes from galectin-1^−/−^ mice are larger and have increased numbers of cells compared with lymph nodes from wild type mice (Fig. 7*B* and data not shown). This difference was significantly amplified when tissue damage resulted from lymphedema. Importantly, one cell population that was dramatically increased in draining lymph nodes from lymphedematous tissue was migratory dermal DCs with the activated phenotype characteristic of iDCs (Fig. 8, *A–C*). This finding is consistent with our *in vitro* finding that galectin-1 in extracellular matrix selectively retarded migration of iDCs (Fig. 3*D*). Thus, the presence of galectin-1 in inflamed tissue may dampen or control the extent of adaptive immune responses by restraining iDC migration from tissue to draining lymph nodes, thus reducing subsequent lymphocyte activation and proliferation in the nodes.

Galectins have pleiotropic functions that depend on the function of the glycoprotein receptors on different cell types that bind specific galectins. Galectins are evolutionarily ancient and have been described in all multicellular organisms, including fungi, nematodes, and insects that all lack an adaptive immune system (132, 133). Interestingly, in mammals, galectin-1 has been found to dampen immune responses in many different systems, independent of the inflammatory insult (72, 134–136). For example, galectin-1 promotes negative selection of auto-reactive thymocytes and promotes differentiation of regulatory leukocyte populations, including tDCs and T_regs_ (33, 36, 37, 92, 93, 101, 137). The inhibitory effect of galectin-1 on leukocyte entry into tissue has been described in several *in vivo* inflammatory settings, including acute peritonitis, contact hypersensitivity, and paw edema models (95, 96). Galectin-1 is constitutively expressed in immune privileged sites such as the eye (138), testes (139), and placenta (140), and galectin-1 expression by tumor cells has been shown to dampen the anti-tumor effect of infiltrating CD8 T cells (141, 142). Galectin-1 kills Th1 and Th17 cells (101, 102) and promotes secretion of the anti-inflammatory cytokine IL-10 (37, 143–145). Galectin-1^−/−^ animals have increased susceptibility to autoimmune diseases, such as experimental autoimmune encephalitis (124), and therapeutic administration of galectin-1 has been effective in models of experimental autoimmune encephalitis, collagen-induced arthritis, and graft *versus* host disease (124, 146, 147). Thus, although one might expect galectin-1 to have diverse effects on mammalian adaptive immunity, depending on the glycoprotein receptors recognized on different cell types, it is remarkable that, *in toto*, galectin-1 has a general immunosuppressive function. Our finding that galectin-1 selectively retards the migration of iDCs both *in vitro* and *in vivo* adds to the roster of immunoregulatory functions for galectin-1 and suggests that some of the immunosuppressive effects of galectin-1 that have been described *in vivo* involve regulation of DC function and migration, as well as direct effects on T cells. Moreover, as has been described for different T cell subsets, the effects of galectin-1 on DCs are determined by the glycan repertoire displayed by different DC subsets, indicating that leukocyte regulation of cell surface glycosylation is a potent mechanism to regulate immune cell function.

## Author Contributions

S. T., M. H. C., B. L., and L. G. B. conceived and coordinated the experiments. S. T., J. H. M., and M. H. C. performed the experiments. S. T. and L. G. B. wrote the manuscript. All authors reviewed the results and approved the final version of the manuscript.

## Footnotes

4 The abbreviations used are:

VEC: vascular endothelial cell
DC: dendritic cell
iDC: immunogenic DC
LEC: lymphatic endothelial cell
ECM: extracellular matrix
tDC: tolerogenic DC
gal: galectin
DTSSP: 3,3′-dithiobis[sulfosuccinimidylpropionate]
PE: phycoerythrin
PFA: paraformaldehyde
pAb: polyclonal antibody
BisTris: 2-[bis(2-hydroxyethyl)amino]-2-(hydroxymethyl)propane-1,3-diol
Bn-α-GalNAc: benzyl-2-acetoamido-2-deoxy-α-d-galactopyranoside
CFSE: carboxyfluorescein succinimidyl ester.
